# Chemically induced oxidative stress affects ASH neuronal function and behavior in *C. elegans*

**DOI:** 10.1038/srep38147

**Published:** 2016-12-06

**Authors:** Eleni Gourgou, Nikos Chronis

**Affiliations:** 1 Department of Mechanical Engineering, University of Michigan, 2350 Hayward Str., Ann Arbor, MI, 48109, USA; 2 Department of Internal Medicine, Division of Geriatric Medicine, Medical School, University of Michigan, 109 Zina Pitcher Place, Ann Arbor, MI, 48109, USA; 3 Department of Biomedical Engineering, University of Michigan, 2200 Bonisteel Blvd., Ann Arbor, MI, 48109, USA

## Abstract

Oxidative stress (OS) impact on a single neuron’s function *in vivo* remains obscure. Using *C. elegans* as a model organism, we report the effect of paraquat (PQ)-induced OS on wild type worms on the function of the ASH polymodal neuron. By calcium (Ca^2+^) imaging, we quantified ASH activation upon stimulus delivery. PQ-treated worms displayed higher maximum depolarization (peak of the Ca^2+^ transients) compared to untreated animals. PQ had a similar effect on the ASH neuron response time (rising slope of the Ca^2+^ transients), except in very young worms. OS effect on ASH was partially abolished in vitamin C-treated worms. We performed octanol and osmotic avoidance tests, to investigate the OS effect on ASH-dependent behaviors. PQ-treated worms have enhanced avoidance behavior compared to untreated ones, suggesting that elevated ASH Ca^2+^ transients result in enhanced ASH-mediated behavior. The above findings suggest a possible hormetic effect of PQ, as a factor inducing mild oxidative stress. We also quantified locomotion parameters (velocity, bending amplitude), which are not mediated by ASH activation. Bending amplitude did not differ significantly between treated and untreated worms; velocity in older adults decreased. The differential effect of OS on behavioral patterns may mirror a selective impact on the organism’s neurons.

Oxidative stress (OS) is one of the most significant types of stress an organism experiences throughout its life. It is the result of exposure to numerous environmental factors, including chemical compounds^1^. The effect of OS on the nervous system is of special interest, since it has been associated with neurodegenerative diseases^2^,^3^, such as Alzheimer’s^4^ and Parkinson’s^5^. Moreover, there is evidence that exposure to certain herbicides, namely paraquat (PQ), known to induce OS^6^, is strongly associated with higher frequency of neurodegenerative diseases in humans^7^.

However, the effect of OS on sensory neurons physiology remains obscure. Altered function of sensory neurons could lead to misperception of the organism’s environment, resulting in a modified behavior, not uncommon in neurodegenerative diseases. A strong correlation between hyperexcitability of sensory neurons and characteristic symptoms (e.g. photophobia in migraine) has been shown^8^, as well as a strong connection between OS and behavioral changes^9^,^10^. Monitoring *in vivo* neuronal function under OS could deepen our understanding on the impact of OS on an organism’s nervous system as well as on the engendered behaviors. Nevertheless, there is a lack of studies exploring *in vivo* the effects of OS on single neuron functionality.

In parallel, *C. elegans* has been broadly used in numerous studies which explore the phenomenon of hormesis, caused, among other harmful conditions, by mild OS^11^. Beneficial effects of low doses of oxidative factors have been reported to result in increased lifespan and increased resistance to other types of stress^12^,^13^. However, the possibility of OS-driven hormetic effects on neuronal Ca^2+^ transients has not been investigated yet.

Here, we investigate the effects of chemically induced OS in the nematode *Caenorhabditis elegans*, using state-of-the-art microfluidic technology and *in vivo* calcium (Ca^2+^) imaging. *C. elegans* has been widely used to investigate both OS and neurodegenerative diseases^14^,^15^ and is therefore an ideal model organism to examine the interplay between OS and neuronal functional physiology^16^,^17^. We exposed wild type worms of different ages to PQ and to vitamin C (VitC), a well-established antioxidant^18^,^19^. We chose PQ concentrations that are known to cause changes in the worms’ physiology due to oxidative damage^16^,^20^, since PQ is known to interfere with electron transfer and catalyze the production of reactive oxygen species (ROS). Using a microfluidic chip^21^, we delivered a hyperosmotic stimulus (glycerol) to the worm’s nose and recorded Ca^2+^ transients from the ASH sensory neuron. We chose ASH, the well-studied worms’ main nociceptor^21^,^22^,^23^,^24^,^25^,^26^,^27^,^28^, because it can be activated by a wide range of repellents^25^,^29^,^30^, thus being especially significant for *C. elegans* survival and environmental perception.

With the exception of very young worms, stimulus-evoked calcium transients from the ASH neuron are elevated in OS-treated worms, when compared to non-treated animals. This effect is partially abolished when the worms are simultaneously treated with PQ and VitC. Octanol avoidance test and glycerol drop assay confirmed that behaviors directly mediated by ASH are enhanced by OS, and this effect is also partially abolished in the presence of VitC. In younger worms, VitC does not reverse the enhancement of the avoidance to glycerol, whereas it reverses the effect on the response to octanol. In contrast, we found that OS-exposed worms did not display significant differences in their locomotive parameters (velocity and bending amplitude) compared to untreated ones, with the exception of the average velocity in older worms.

We conclude that exposure to chemically induced OS significantly affects the function of ASH neuron, as well as avoidance behaviors directly mediated by ASH. The observation that behaviors independent of ASH are not correspondingly affected, could strongly suggest that neurons are not uniformly affected by chemically induced OS. This could further imply a differential susceptibility to OS among neurons, neuronal circuits and subsequently, behavioral patterns. Our results can pave the way for further experiments on the interplay between OS and neuronal function.

Last, we suggest that calcium imaging and microfluidic platforms can be a powerful tool for revealing OS-dependent changes in neuronal function in *C. elegans*, and potentially in other model organisms as well.

## Results

### PQ treatment increases the magnitude of ASH Ca^2+^ transients

**Figure 1:**
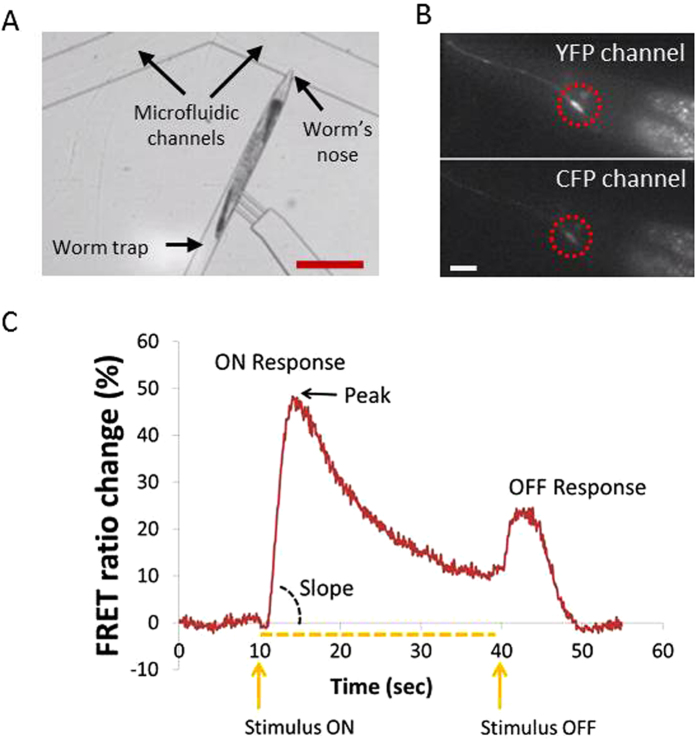
(**A**) The 4-flow microfluidic chip is used for immobilizing single worms and delivering a chemical stimulus (glycerol) to their nose^21^ to induce neuronal activation. Before and after the delivery of the stimulus, a control buffer solution is being delivered to the worm’s nose. Scale bar: 200 μm. (**B**) Fluorescence image of a trapped worm. The ASH neuron (highlighted by the dotted circle) is labelled with the FRET indicator TNXL. Top Image: YFP channel, bottom image: CFP channel, scale bar: 5 μm. (**C**) A typical Ca^2+^ transient response of the ASH neuron. The stimulus is delivered at 10 sec and removed at 40 sec. The maximum FRET ratio change upon delivery of the stimulus is considered as the “peak” response. The ASH neuron has a biphasic (ON/OFF) response^21^.

**Figure 2:**
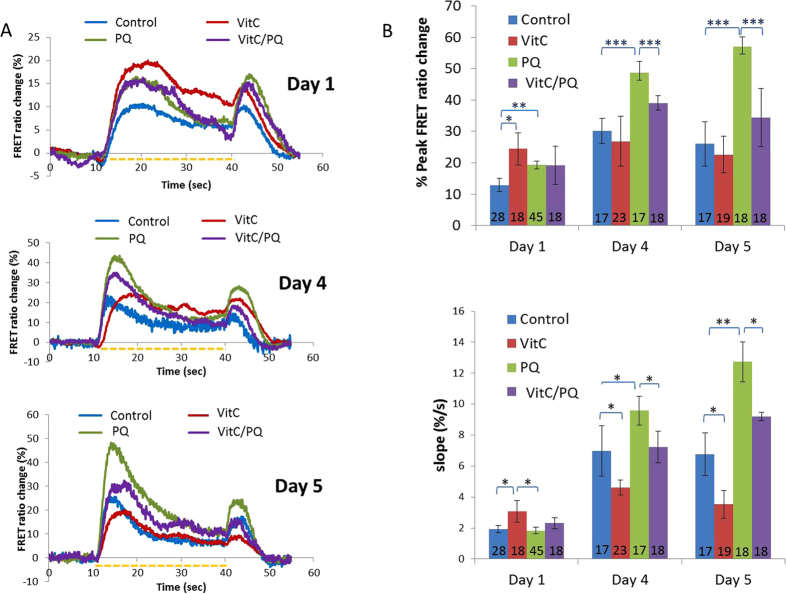
(**A**) Ratiometric calcium transients of the ASH neuron upon delivery of hyperosmotic stimulus (1 M glycerol) at 3 different ages (L4 animals are considered as Day 0 worms). Worms studied: untreated (control worms), VitC worms (grown at the presence of 1 mM vitamin C), PQ worms (grown at the presence of 0.1 mM PQ) and VitC/PQ worms (grown at the simultaneous presence of 1 mM vitamin C and 0.1 mM PQ). The dashed line represents the presence of the stimulus. (**B**) The maximum peak and rising slope of the on-response (calculated from A). The number in each column represents the number of worms tested. Error bars indicate standard error of mean. ***p < 0.001, **p < 0.01, *p < 0.05 (Student’s t-test).

We used the microfluidic platform to quantify the effect of rearing on 0.1 mM PQ to the function of the ASH neuron (Fig. 1). We monitored the activity of ASH using the TNXL indicator in response to a 30 sec stimulus pulse (1 M glycerol). We examined four groups of age-synchronous worm populations: treated with 0.1 mM PQ, treated with 1 mM VitC, treated with 0.1 mM PQ + 1 mMVitC and untreated (control) ones (Fig. 2A). We imaged a total of 256 worms, aged L4 + 1 day (marked here as “Day 1” worms), L4 + 4 days (referred to as “Day 4” worms) and L4 + 5 days (mentioned here as “Day 5” worms).

The average peak and the slope of the ON response (Fig. 1) in animals previously treated with PQ increased in all days studied, compared to the control animals, with the exception of Day 1 rising slope (Fig. 2B and Table 1). Comparing the peak and the slope of the rising phase between the PQ-treated animals and worms treated with PQ + VitC, we find that in Days 4 and 5 simultaneous treatment with PQ + VitC results in bringing calcium (Ca^2+^) transients back to the level of control animals of the same age. However, this is not the case in Day 1 worms, where concurrent treatment with PQ and VitC has the same effect as PQ alone. Interestingly, VitC has no effect on the peak of Ca^2+^ transients, compared to Day 4 and Day 5 untreated worms (Fig. 2B). However, in Day 1 worms, VitC treatment increases both the peak and the slope of the FRET % change, whereas the slope is reduced in Day 4 and Day 5 worms.

When delivering PQ instead of glycerol to the nose of untreated animals, no ASH activation was observed (suppl. Fig. 1).

### PQ treatment enhances ASH mediated behavior

#### Osmotic avoidance

We examined whether the observed OS-related changes in ASH function result in modified ASH-mediated worm behavior. To this end, as a direct read out of ASH activation, we assayed osmotic avoidance, by running the glycerol drop test.

**Figure 3:**
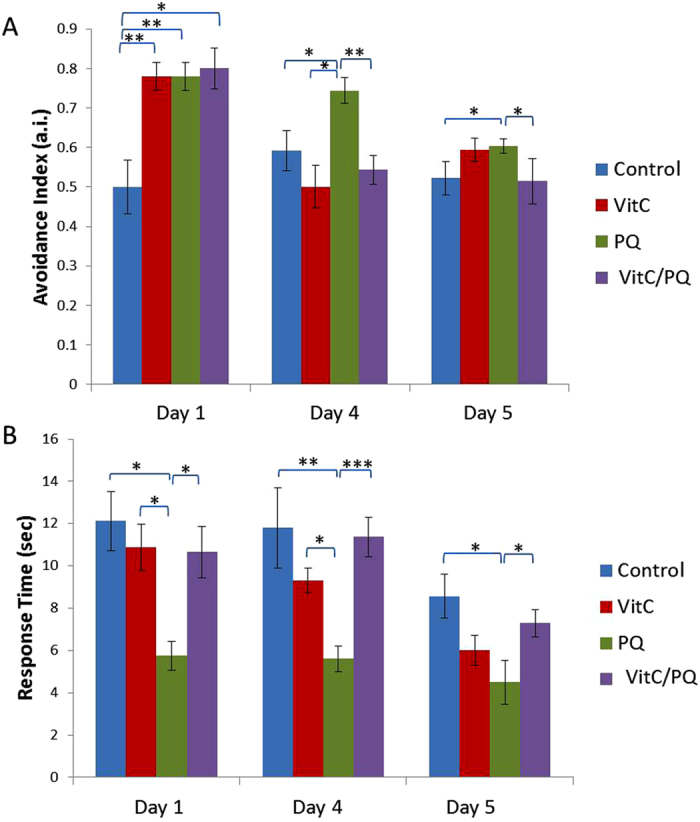
Avoidance index and response time to 0.5 M glycerol drop test (**A**) and 30% octanol (**B**) respectively in worms of four studied groups (Control: untreated animals, VitC: worms grown at the presence of 1 mM vitamin C, PQ: worms grown at the presence of 0.1 mM PQ, VitC/PQ: worms grown at the simultaneous presence of 1 mM vitamin C and 0.1 mM PQ), for three different ages (L4 animals are considered as Day 0 worms). (**A**) Avoidance index (a.i.) to glycerol changes after assaying 20 worms, 5 trials/worm for each studied group. (**B**) Response time to octanol differentiations after assaying 20 worms from each studied group. Error bars indicate standard error of mean. ***p < 0.001, **p < 0.01, *p < 0.05 (Student’s t-test).

Our results show (Fig. 3A and Table 1) that treatment with PQ increases the avoidance index (a.i.) in all days studied (56% for Day 1, 25% for Day 4 and 15% for Day 5). Simultaneous exposure of the worms to PQ and VitC results in reversing the PQ effect during Days 4 and 5, but not on Day 1. The VitC rescuing effect on the osmotic avoidance is aligned with the effect of VitC on ASH Ca^2+^ transients, where the amplitude of the ion influx after concurrent exposure to VitC and PQ hovers around the control levels during Days 4 and 5, but not on Day 1 (Figs 3A and 2B, top).

#### Octanol assay

We tested worms’ reaction time to the repellent odor of octanol, a behavior known to be mediated by ASH. The aversive response to 30% octanol is found to be significantly faster in worms exposed to PQ during their life (Fig. 3B and Table 1) in all days studied. Indeed, the time needed for a worm to initiate a reversal is decreased by 52% in Days 1 and 4, and by 47% in Day 5, showing almost twice as fast a response in all days examined (Fig. 3B). Simultaneously administering VitC and PQ, appears to restore response time by 88% at Day 1, by 96% at Day 4 and by 85% at Day 5, showing that the presence of the antioxidant has indeed a rescuing effect on PQ-induced impact. Vitamin C alone does not have any effect on the octanol avoidance response.

### Exposure to PQ reduces older worms mean velocity, but does not affect other locomotion parameters

**Figure 4:**
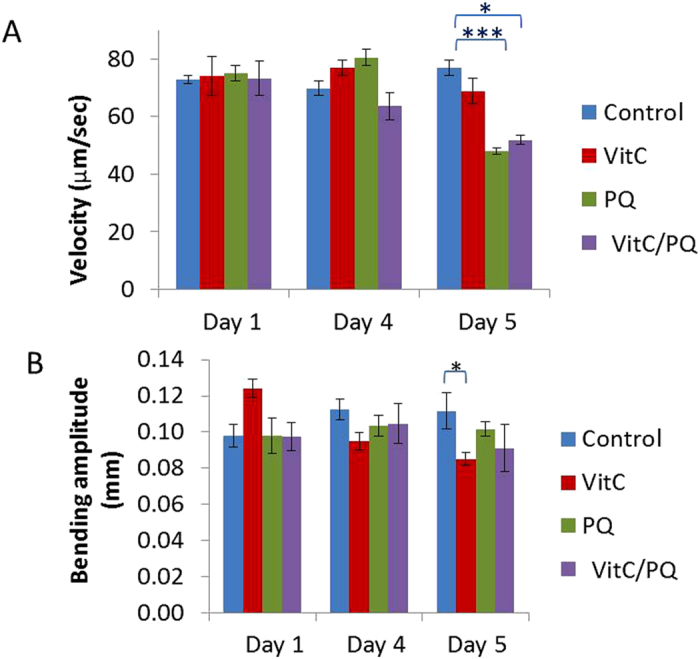
Comparison of velocity (**A**) and bending amplitude (**B**) between untreated (control) and treated worms of three different ages (Control: untreated animals, VitC: worms grown at the presence of 1 mM vitamin C, PQ: worms grown at the presence of 0.1 mM PQ, VitC/PQ: worms grown at the simultaneous presence of both 1 mM vitamin C and 0.1 mM paraquat). 18–22 worms were tracked from each studied group. Error bars indicate standard error of mean. ***p < 0.001, *p < 0.05 (Student’s t-test).

By using a custom-made tracking system^31^,^32^,^33^, we analyzed worms’ locomotion parameters (mean velocity, bending amplitude). It is shown that treatment with PQ results in decreased velocity (μm/sec) in animals of Day 5, by 38% (control animals mean velocity = 77 μm/sec, PQ-treated animals mean velocity = 48 μm/sec) (Fig. 4A and Table 1). No significant change was detected, however, for Days 1 and 4. It is also shown that, in Day 5, simultaneous treatment with PQ and VitC results in reduced velocity, as well (PQ/VitC-treated animals mean velocity = 52 μm/sec; 32% decrease, compared to control animals) (Fig. 3A). As far as it concerns treatment with VitC alone, no statistically significant change was detected in any of the examined days.

When analyzing the bending amplitude (Fig. 4B and Table 1), the only effect observed is a significant drop between untreated and VitC-treated Day 4 animals on Days 4 and 5 (VitC-treated animals bending amplitude = 0.10 mm compared to control Day 4 animals bending amplitude = 0.12 mm, and VitC-treated Day 5 animals bending amplitude = 0.09 mm *versus* control Day 5 animals bending amplitude = 0.12 mm). However, this difference is of borderline statistical significance, since the *p*-value is exactly 0.05.

## Discussion

Paraquat (PQ) is well-known to induce OS in living organisms^34^, including *C. elegans*^16^,^35^. Additionally, the hormetic effect of low intensity PQ-induced OS has been well articulated in *C. elegans*^36^,^37^, as it has been reported that mildly elevated ROS levels through treatment with PQ can actually promote longevity^16^,^36^,^38^.

Here we show for the first time that previous exposure to chemically induced OS affects both the magnitude and the time rate of the Ca^2+^ influx which takes place upon stimulus delivery, at ASH sensory neuron. Specifically, our results reveal that in young worms (Days 1, 4 and 5 of adult life), treatment with a sublethal concentration of PQ leads to elevated ASH Ca^2+^ transients, compared to untreated animals, when stimulated by hyperosmotic solution. The observed ASH hypersensitivity (as quantified by the maximum peak and rising slope of the Ca^+^ transients upon delivery of the stimulus) increases the longer the worms are exposed to OS (Fig. 2). This could be attributed to the effect of accumulated ROS as a result of sustained exposure to PQ^39^,^40^ or to age-related deterioration of the antioxidant mechanisms^41^,^42^. Moreover, it is known that Ca^2+^ influx in ASH depends on transient receptor potential channels (TRPV) and voltage-gated ion channels (VGCC)^27^,^43^, the physiology of which is age-dependent^44^,^45^. Therefore, a potential explanation for age-related, OS-induced Ca^2+^ transients modifications^21^ could be that the abovementioned channels are affected by chemically induced OS more as the worms age. Interestingly, alterations in Ca^2+^ transient rising slope do not always follow the changes in the transients’ maximum peak, as is the case of Day 1 worms, where PQ raises the maximum peak value but has no effect on the slope. Possibly, the molecular components of the Ca^2+^ channels^46^, responsible for the rate and the magnitude of the influx, do not get uniformly affected by OS. However, the opposite is observed when administering vitamin C alone, since in worms of Days 4 and 5 the latter does not affect the maximum peak of the transient, but it results in smaller slope. This finding could be indicative of the opposite ways PQ and vitamin C act on the Ca^2+^ influx mechanism.

The increase in the amplitude and rate of stimulus-evoked ASH Ca^2+^ transients could mark an OS-caused enhanced reaction to environmental cues. Indeed, PQ-exposed worms have a stronger avoidance response against the hyperosmotic solution and they respond faster to octanol in all days examined, compared to control animals (Fig. 3). These findings could be interpreted in the context of mild OS hormetic effect^47^, resulting in a potentially advantageous enhancement of physiological mechanisms and behaviors. Moreover, since substances with hormetic effects typically prolong the worm’s lifespan^11^,^12^,^13^, it is possible that treatment with PQ results in ASH Ca^2+^ transients comparable to the ones of untreated, younger animals. Furthermore, since PQ has been shown to increase lifespan^16^,^36^,^38^, we can speculate that other life-prolonging agents might have a similar effect on ASH Ca^2+^ transients. Further experiments would be necessary to clarify whether PQ acts through ASH-specific or global mechanisms.

Findings regarding both glycerol drop test and octanol avoidance seem to be lined up more with changes in the magnitude than the rate of the Ca^2+^ influx response, especially in Day 1 worms. This could mean that the behaviors examined depend more on the total amount of Ca^2+^ ions than on the rate of the influx, at least for younger adults.

Intracellular ascorbic acid (vitamin C) serves several protective functions, as it scavenges radical species before they can damage DNA, proteins, or lipids^48^, and it contributes in recycling other antioxidants^48^. The established antioxidant role of vitamin C^18^,^49^, has not been really proven when it comes to *C. elegans*^50^. This is partially due to conflicting reports about its impact on *C. elegans* lifespan^37^,^51^,^52^. Our results contribute to this discussion, showing for the first time that when offered concurrently with the oxidative factor, it can lead to abolishing OS implications. Importantly, the reversing effect of vitamin C in PQ-exposed worms applies to both the ASH Ca^2+^ transients and to ASH-depended behaviors. High ascorbate concentration in neurons is thought to be generated and maintained due to specific transporters^53^, the function of which, in relation to aging, is under investigation^54^. The existence of a similar transporter in *C. elegans* could offer a possible explanation as to why the rescuing effects of vitamin C are more intense in older worms.

Interestingly, vitamin C alone increases Ca^2+^ influx in the ASH neurons of Day 1 worms, and also affects ASH controlled behaviors (Figs 2 and 3). It has been found, in human embryonic cells, that the number of TRPV1 channels increases due to vitamin C impact^55^. Since calcium influx in ASH depends on both TRPV and VGCC channels^44^,^45^, we can assume that the vitamin C effect might possibly occur because of changes in the TRPV channels abundance on ASH membrane. It is noteworthy that Vitamin C alone decreases slope in days 4 and 5 (Fig. 2B, bottom); however, it does not decrease the peak of % FRET change (Fig. 2B, top), which accounts for the magnitude of the response. As the slope represents the rate by which the Ca^2+^ enter the cell, we can assume that Vitamin C has an effect exactly on this part of the Ca^2+^ influx controlling molecular mechanism. The fact that Vitamin C reduces that rate whereas PQ increases it and the two combined have an intermediate effect, could mean that the two compounds act antagonistically. If this is true, then Vitamin C does not “undo” whatever effect PQ has; however, it still results in abolishing this effect. Future experiments that will shed light on the exact mechanism by which Vitamin C acts would be of special interest. The possibility of Vitamin C itself having a hormetic effect could be further examined, as well.

It has been reported that mutants with increased oxidative stress show reduced motor activity^56^ and that exposure to peroxide stress causes loss in mobility^39^. These findings are in agreement with our results, which unveil a decrease in average velocity in PQ-treated worms on Day 5. This decrease is not reversed by vitamin C, suggesting that the amount of vitamin C used may not be enough to invert the effect. More importantly, velocity depends on a number of neurons, not including ASH, which may be affected differently by PQ-induced OS.

The actual finding that avoidance behaviors and locomotion parameters are affected differently by chemically-induced OS, could suggest that this also applies to the neurons involved in the respective behavioral expressions. Neuron-specific induction of OS has been reported in the past^57^,^58^, and cell specific susceptibility to OS has been observed^59^,^60^. Possibly, the developmental stage of both PQ and vitamin C administration, the type of OS (chemically induced), and importantly, the dose of PQ used, might not affect in the same extent all neurons in the worms’ nervous system. What is more, vitamin C transmembrane transporters seem to display themselves a type of cell-specificity, supporting further the above assumption^61^. Hence, we claim that our results can be considered as a potent indication towards a type of neuron-specific sensitivity when it comes to chemically induced oxidative stress.

## Methods

### *C. elegans* strains and treatment

We used an integrated line that expressed the TN-XL calcium indicator in the ASH neuron, under the *sra-6* promoter [NKC2311 micIs211(p_*sra-6*_::TNXL)]. We integrated the line AQ20801jEx211[p_*sra-6*_::TN-XL] by using a standard UV exposure protocol, as described previously^21^. In all of our experiments, we used age-synchronized hermaphrodite worms^62^ that were grown at 20 °C on OP50-seeded agar plates. For treating the worms with PQ, VitC and PQ + VitC, synchronized populations were grown on seeded NGM plates containing 0.1 mM PQ, 1 mM VitC and 0.1 mM PQ/1 mM VitC^16^ respectively. When conducting the Ca^2+^ imaging experiments, animals were imaged within 10 min after removal from the treatment plates.

### Microfluidic chip and fluidic setup

Soft lithography was used to fabricate the PDMS/glass microfluidic chip. The fluidic setup was calibrated and controlled through a custom-made LabView algorithm as described previously^21^.

In all calcium imaging experiments, the stimulus was delivered on the 10^th^ sec of the recording period and was withdrawn on the 40^th^ sec of the recording period (Fig. 1) (the presence of the stimulus is marked on the plots with a dotted line). The length of the stimulus was chosen based on previous experiments of our group (see also Chokshi *et al*., 2010).

### FRET analysis, peak and slope calculations

#### Image and data analysis

We used a custom made LabVIEW program to extract the mean FRET ratio from each fluorescent image we acquired (Chokshi *et al*., 2010). Briefly, each fluorescent image was split by a dual imager into two separate images, one representing the YFP (yellow fluorescent protein) and one the CFP (cyan fluorescent protein) channels. The LabVIEW program required four pieces of initial information, specified by the user for each examined worm: (1) the region of interest, defining the boundary of the neuronal soma, in the YFP channel, (2) the region within which the neuron moves while the animal is immobilized in the microfluidic channel, in the YFP channel (3) the region defining the background fluorescence of the worm, in the YFP channel and (4) the initial coordinates of the neuron in both YFP and CFP channels. The LabVIEW program first calculates the difference between the initial coordinates of the neuron’s center in each channel. It uses this difference to correct for any misalignment between the YFP and CFP images. It then tracks the neuron of interest in each channel and extracts the corresponding background-subtracted, mean fluorescence intensity. The FRET ratio is calculated as the ratio of mean fluorescence intensity of the neuron in the two channels.

#### FRET ratio % change, peak and slope calculations

For the FRET ratio % change, the average fluorescence ratio in a 3 second window (t = 1–4 s) was set as the baseline. The % change in fluorescence intensity for the region of interest was plotted for all image stacks (Chalasani *et al*., 2007). The “peak” refers to the maximum value of the FRET ratio % change as observed during the ON response of the ASH neuron (Fig. 1). The “slope” refers to the rising slope of the ON response, and is calculated as the maximum value of the FRET ratio change observed (% peak FRET ratio change), over the time needed to reach this maximum, counting from the stimulus onset. We have selected the peak and rising slope of the ON response since this peak is indicative of the total amount of calcium entering the cell and consequently accounts for the amplitude of the cell’s response, whereas the rising slope corresponds to the calcium ions influx.

### Behavioral assays

#### Octanol Avoidance Assay

We used 30% (v/v) 1-octanol solution (freshly prepared daily, diluted in 100% ethanol) to assay the octanol avoidance behavior of OS-exposed worms. Octanol avoidance in this concentration range is mediated solely by the ASH neurons^63^,^64^. Fresh NGM plates, used as intermediate and assay plates were prepared on the day of the experiment. Worms were picked from their OP50 growing plates and transferred to new (intermediate) plates, left for 1 min, and then were transferred into the assay plates, where they were tested after 5 min^64^. Intermediate and assay plates did not contain PQ or VitC and they were not seeded with bacteria. The blunt end of an eyelash hair was dipped in the octanol solution and was placed in front of the nose of a forward moving animal. The time required by the animal to initiate backward movement was recorded using a timer^63^. Octanol avoidance assays were conducted on well-fed worms, as feeding status may affect the response of animals to octanol^63^. Animals were tested on different days, at least 5 worms on each day. In total, ~20 animals were examined for each experimental condition.

#### Glycerol Drop Test

The drop assay was performed following a previously described procedure^65^. A drop of 0.5 M glycerol (freshly prepared daily, dissolved in 30 mM Tris, 100 mM NaCl, 10 mM KCl; M13 buffer) was placed on the agar surface, near the tail of a forward moving animal. The drop quickly spread out surrounding the animal and finally reached the anterior amphid sensory neurons. Animals’ response to the osmotic stimulus resulting in initiating a backward motion was scored as a positive response, when the worm responded within 5 sec. Drop tests were conducted on unseeded NGM plates and drops were delivered using custom-made glass capillaries (pulled by hand on a flame to make them narrower). On the other end of the capillary, a piece of polyethylene tubing was mounted and connected to a 5 ml syringe through a lauer. The avoidance index (a.i.) was calculated by dividing the number of positive responses to the total number of trials^65^ and the mean a.i. was calculated at the end of each experiment. All groups were tested using single worm assays, where each worm’s response was tested by applying 5 drops, with a 5 min interval between successive trials. 8–10 individual, well-fed animals per group were tested each day, for 2–3 days, giving a total of ~20 worms for each group of interest.

### Worm Tracking

Worms were tracked to analyze their locomotion behavior on NGM plates using an automated worm tracking system developed in Xu Lab, Life Sciences Institute, University of Michigan, as previously described^31^,^32^,^33^. Briefly, NGM plates were seeded with a thin layer of fresh bacterial lawn (OP50) 10 min prior to tracking. Tracking was performed on freely moving worms, at ~20 °C and at a relative humidity of ∼40%, with the petri dish lid off. The tracking system includes a stereomicroscope (Zeiss Stemi 2000 C) mounted with a digital camera (Cohu 7800) and a digital motion system (Parker Automation) that follows worm movement, as well as a custom made software package. To record locomotion, worm images were captured at 2 Hz for 2 min and the mean velocity (centroid displacement per second) of each worm was quantified using laboratory-developed software. The motion data were also compressed, integrated and stored as a multimedia file format (AVI). The extent of body bending (bending angle) was calculated as described previously^33^,^66^ using binary worm images, thinned to obtain the “skeleton image” of the worm and broken into equal-length segments.

### Statistical Analyses

Statistical analyses were performed using GraphPad Prism 6 (GraphPad Software Inc., La Jolla, CA, USA) and Excel (Microsoft, Redmont, WA). Results are presented as the mean ± standard error. Imaging data, octanol avoidance and glycerol drop test results as well as worm tracker derived data were tested by unpaired two-tailed Student’s *t*-test and all differences were considered statistically significant at *p*-value < 0.05 (* indicates p < 0.05, ** indicate p < 0.01, *** indicate p < 0.001). Grubb’s outlier’s test was used in the processing of the behavioral assays results.

## Additional Information

**How to cite this article**: Gourgou, E. and Chronis, N. Chemically induced oxidative stress affects ASH neuronal function and behavior in *C. elegans. Sci. Rep.* **6**, 38147; doi: 10.1038/srep38147 (2016).

**Publisher's note:** Springer Nature remains neutral with regard to jurisdictional claims in published maps and institutional affiliations.

## Supplementary Material

Supplementary Information

## Figures and Tables

**Table 1:** Summary of the effect of each treatment to the examined metrics compared to untreated (control) worms for Day 1, 4 and 5 worms.

		Treatment & Day (d) studied								
		PQ			VitC			PQ & VitC		
METRICS		d1	d4	d5	d1	d4	d5	d1	d4	d5
**ASH function (calcium imaging)**	Peak	*50 ± 27	***61 ± 39	***118 ± 49	*90 ± 51	−11 ± 34	13 ± 29	49 ± 54	30 ± 31	31 ± 46
	Slope	5 ± 15	*37 ± 43	**88 ± 42	*58 ± 40	*−33 ± 21	*−48 ± 17	19 ± 23	3 ± 29	35 ± 20
**Avoidance Index** (glycerol drop test)		**56 ± 20	*25 ± 12	*15 ± 9	***56 ± 20	−15 ± 11	13 ± 10	**60 ± 22	−8 ± 9	−2 ± 12
**Response time** (octanol avoidance)		***−52 ± 68	**−52 ± 9	*−47 ± 14	−10 ± 14	−21 ± 14	−30 ± 12	−12 ± 14	−3 ± 17	−15 ± 13
**Locomotion parameters**	-velocity	3 ± 4	15 ± 6	***−37 ± 3	1 ± 10	10 ± 6	−10 ± 7	0.5 ± 8	−8 ± 8	*−32 ± 3
	-bending amplitude	0 ± 11	−9 ± 6	−9 ± 9	20 ± 9	−18 ± 5	*−27 ± 7	0 ± 9	−18 ± 10	−9 ± 12

Results are given as % change compared to untreated worms. Error is calculated based on error propagation analysis. Values have been rounded to the nearest integer. Cells without stars indicate p > 0.05 (non-significant difference), one star (*) indicates p < 0.05, two stars (**) indicate p < 0.01, and three stars (***) indicate p < 0.001 (Student’s *t*-test) (see also Suppl. Table S1).
