# Supplementary material for: Chemically induced oxidative stress affects ASH neuronal function and behavior in C. elegans
Source: Sci Rep. 2016 Dec 6;6:38147. doi: 10.1038/srep38147 (PMC5138595; doi:10.1038/srep38147)
Supplement: Supplementary Information [file srep38147-s1.pdf]

Chemically induced oxidative stress affects ASH neuronal function and behavior in *C. elegans*

Eleni Gourgou[1, 2], Nikos Chronis[1, 3, +]

[1]Department of Mechanical Engineering, University of Michigan, Ann Arbor, MI, USA.
[2]Department of Internal Medicine, Division of Geriatric Medicine, Medical School, University of Michigan, Ann Arbor, MI, USA
[3]Department of Biomedical Engineering, University of Michigan, Ann Arbor, MI, USA

+corresponding author, Nikos Chronis: chronis@umich.edu

SUPPL. FIGURE 1

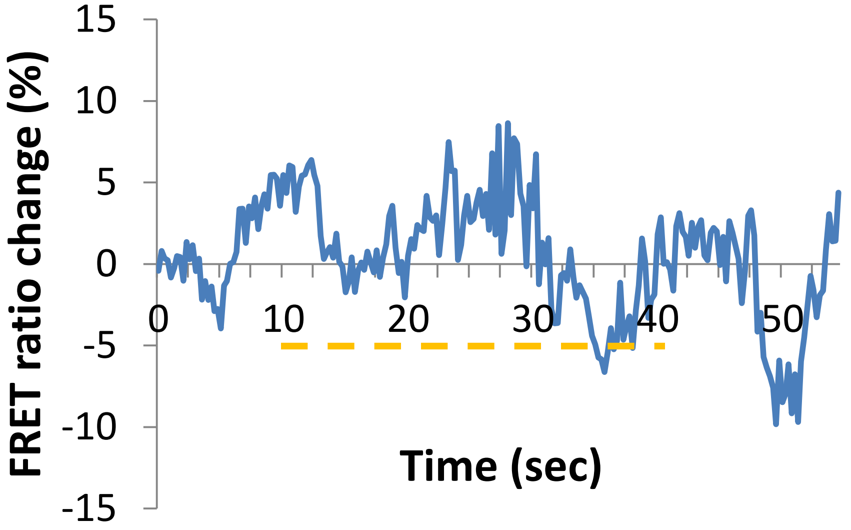

Suppl. Fig. S1: Ratiometric calcium transients in the ASH neuron, upon delivery of paraquat (0.1mM PQ), in Day 4 worms. Curve represents an average of 10 recordings. The dashed line represents the presence of the stimulus.

SUPPL. TABLE 1

METRICS		Treatment & Day (d) studied								
		PQ			VitC			PQ & VitC		
		d1	d4	d5	d1	d4	d5	d1	d4	d5
**ASH function** (calcium imaging)	peak	0.042	0.0003	0.0003	0.029	0.062	0.35	0.10	0.08	0.6
	slope	0.76	0.02	0.008	0.03	0.01	0.047	0.24	0.89	0.46
**Avoidance Index** (glycerol drop test)		0.002	0.03	0.028	0.001	0.24	0.28	0.002	0.50	0.91
**Response time** (octanol avoidance)		0.001	0.002	0.03	0.49	0.15	0.12	0.43	0.81	0.31
**Locomotion parameters** -velocity		0.87	0.16	0.0001	0.63	0.43	0.30	0.45	0.47	0.05
-bending amplitude		0.63	0.28	0.16	0.08	0.058	0.05	0.62	0.59	0.28

Suppl. Table S1: Summary of p-values (Student's *t*-test) for each treatment and the examined metrics, compared to untreated (control) worms for Day 1, 4 and 5. Values have been rounded to the nearest significant decimal. Uncolored cells indicate p>0.05 (non-significant difference), yellow cells indicate p<0.05, green cells indicate p<0.01, and blue cells indicate p<0.001.

SUPPL. FIGURE 2
(A) Day 1

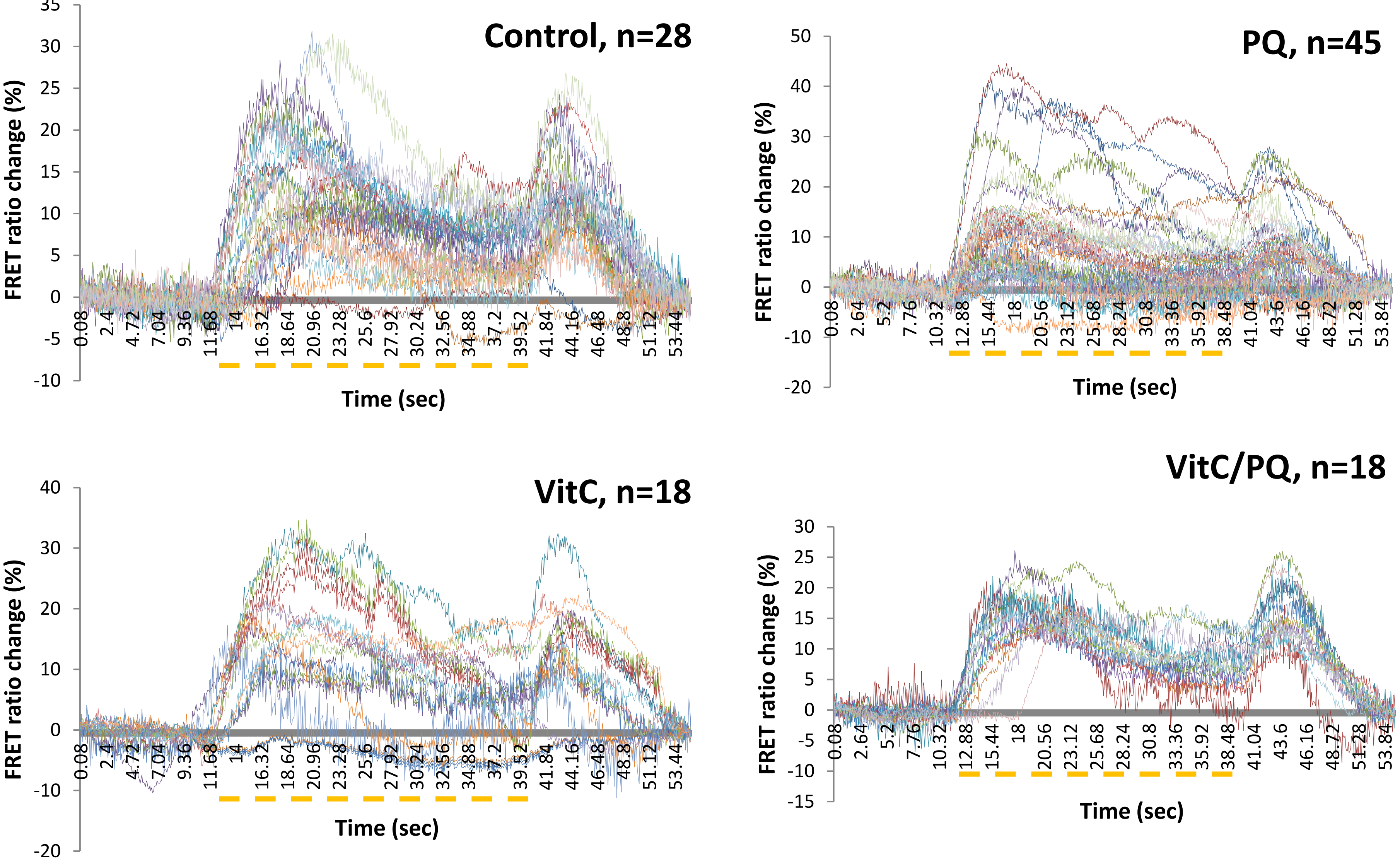

SUPPL. FIGURE 2
(B) Day 4

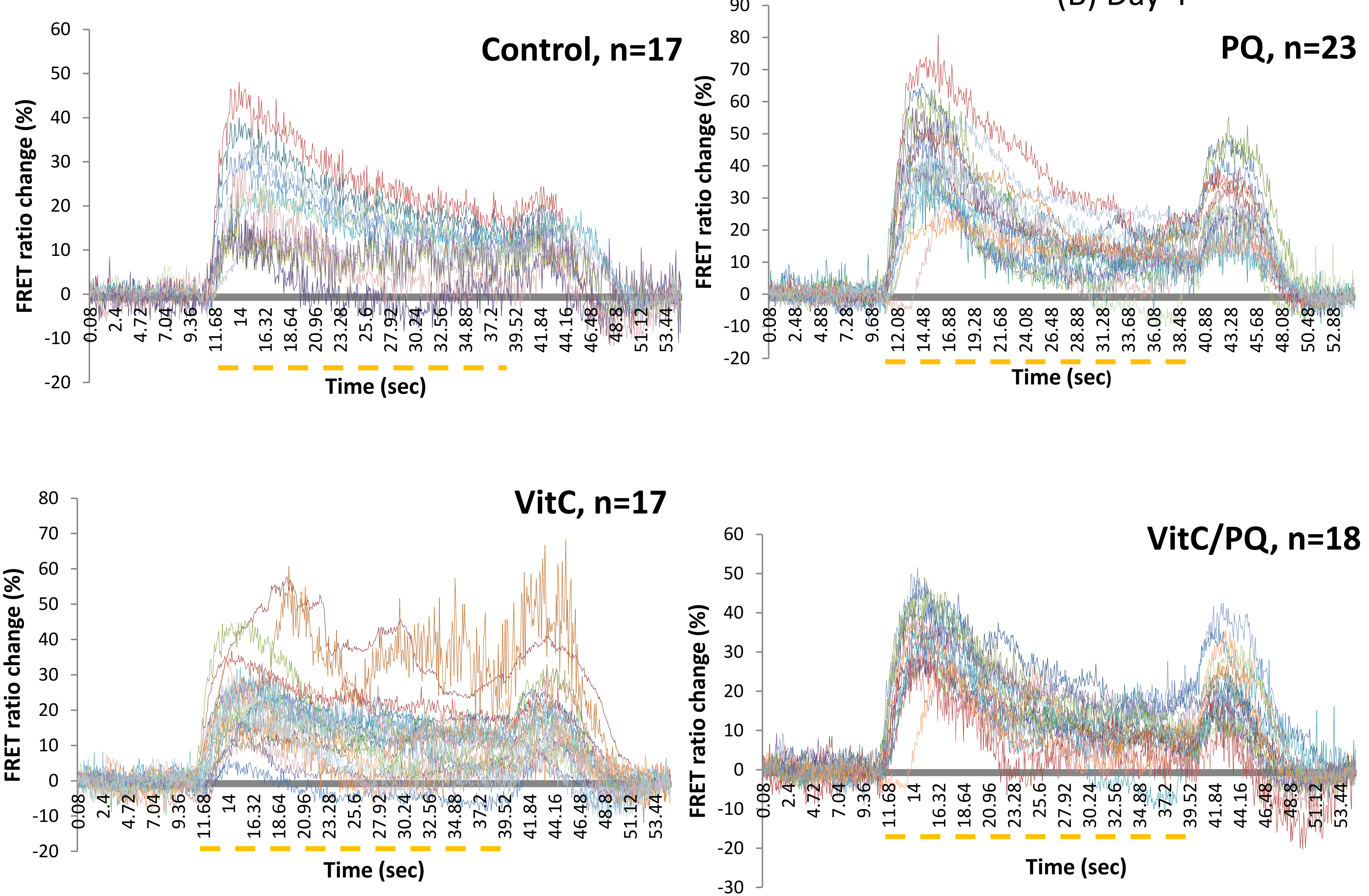

SUPPL. FIGURE 2
(C) Day 5

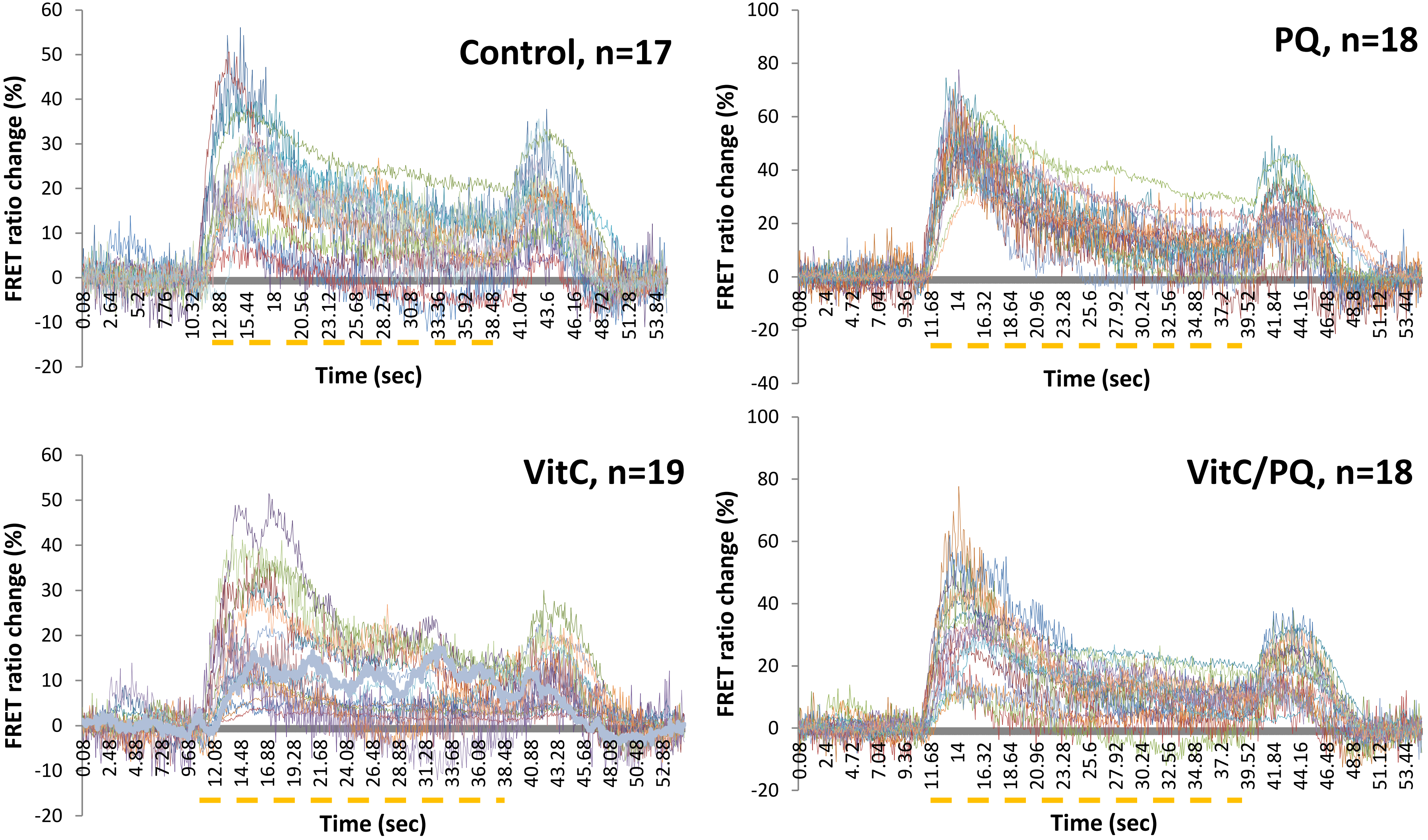

Suppl. Figure S2: Individual ratiometric calcium transients of the ASH neuron, upon delivery of hyperosmotic stimulus (1M glycerol), in worms of four studied groups (Control: untreated animals, VitC: worms grown at the presence of 1mM vitamin C, PQ: worms grown at the presence of 0.1mM PQ, VitC/PQ: worms grown at the simultaneous presence of both 1mM vitamin C and 0.1mM PQ). (A) Day 1 recordings, (B) Day 4 recordings, (C) Day 5 recordings (L4 animals are considered as Day 0 worms), n= number of worms tested. The dashed line represents the presence of the stimulus.

SUPPL. TABLE 2, Octanol Response Time

	RESPONSE TIME (sec)													
	Day1					Day4					Day5			
Worm	Control	PQ	VitC	VitCPQ		Control	PQ	VitC	VitCPQ		Control	PQ	VitC	VitCPQ
1	12.83	6.75	7.5	9.82			3.48	10.57	11.67		13	4	8	9
2	15.86	5.31	13.12	10.11		15.72	3.84	10.94			6	5	5	8
3	14.15	2.7	8.58	6.7		16.84	6.22	10.17	15.72		7	7	5	5
4	12.95	5.4	12.75	13.47		10.42	7.84	6.58	13.42		7	2	6	8
5	8.75	6.7	13.8	12.54		7.55	7.58	8.17	9.54		12	7	5	5
6	18.7	7.45	13.61	14.13		8.48	6.42	11.46	12.27		8	4	6	7
7	12.9	3.46	11.74	16.03		11.78	4.41	8.22	14.52		7	2	11	9
8	7.71	8.25	5.89	5.43		14.83	3.27	8.37	10.74		8	4	7	5
9	5.27	7.69	6.6	7.65		17.75	7.35	11.46	6.45		9	5	6	8
10	11.82	4.28	14.23	8.71		9.54	4.52	9.98	8.85		7	5	5	5
11	16.84	3.81	7.61	11.23		8.67	2.75	11.22	7.42		11	4	12	11
12	15.14	6.41	13.64	5.1		5.39	7.15	9.98	14.52		13	6	6	5
13	11.91	5.68	14.2	14.46		10.79	6.74	7.21	15.6		6	4	11	7
14	9.71	8.54	12.73	11.65		12.85	8.61	12.52	7.51		7	5	5	8
15	17.9	2.56	12.61	15.23		12.7	5.33	7.31	14.46		7	7	6	8
16	11.8	7.22	4.98	15.14		14.7	6.52	9.48	8.82		12	2	10	9
17	8.8	5.7	11.8	6.33		10.2	2.38	8.33	12.3		8	2	3	5
18	4.1	4.78	12.7	6.65		8.3	8.44	10.29	6.39		7	7	4	12
19	12.8	6.64	9.64	9.56		12.05	6.1	15.29	11.4		7	3	8	8
20	12.1	5.71	8.8	11.74		11.01	4.7	9.17	11.15		12	5	3	7

Suppl. Table S2: Response time to 30% octanol in worms of four studied groups (Control: untreated animals, VitC: worms grown at the presence of 1 mM vitamin C, PQ: worms grown at the presence of 0.1mM PQ, VitC/PQ: worms grown at the simultaneous presence of 1 mM vitamin C and 0.1 mM PQ), for three different ages (L4 animals are considered as Day 0 worms). Response time in sec, 20 worms assayed from each studied group.

SUPPL. TABLE 3, Glycerol Avoidance Index, (A) Day1

	**Day1**																							
Worm	Control					A.I.	PQ					A.I.	VitC					A.I.	VitCPQ					A.I.
1	Y	Y	N	Y	N	0.6	Y	Y	Y	N	Y	0.8	Y	Y	Y	Y	Y	1	Y	Y	Y	Y	Y	1
2	Y	N	Y	N	Y	0.6	Y	Y	Y	N	Y	0.8	Y	Y	N	Y	Y	0.8	N	Y	Y	N	Y	0.6
3	Y	Y	Y	Y	N	0.8	N	Y	Y	Y	Y	0.8	Y	N	Y	N	Y	0.6	Y	Y	N	Y	Y	0.8
4	N	Y	N	N	N	0.2	Y	Y	N	Y	Y	0.8	Y	Y	Y	Y	N	0.8	Y	Y	Y	N	Y	0.8
5	N	N	N	Y	Y	0.4	N	Y	Y	Y	Y	0.8	Y	Y	N	N	Y	0.6	Y	N	Y	Y	N	0.6
6	N	N	Y	N	Y	0.4	Y	Y	Y	Y	N	0.8	N	Y	Y	Y	Y	0.8	Y	Y	Y	Y	Y	1
7	N	Y	Y	Y	N	0.6	Y	N	Y	N	Y	0.6	Y	Y	Y	Y	N	0.8	N	Y	Y	N	Y	0.6
8	Y	Y	N	Y	Y	0.8	Y	Y	Y	Y	Y	1	Y	N	Y	Y	Y	0.8	Y	Y	Y	Y	Y	1
9	N	N	Y	N	N	0.2	Y	N	Y	Y	Y	0.8	N	Y	Y	Y	Y	0.8	N	Y	Y	Y	N	0.8
10	Y	N	N	Y	N	0.4	N	Y	Y	Y	N	0.6	Y	Y	N	Y	Y	0.8	Y	Y	Y	N	Y	0.8
11	N	Y	Y	Y	N	0.6	Y	Y	N	Y	Y	0.8	N	Y	Y	Y	Y	0.8	Y	N	Y	Y	N	0.6
12	Y	N	N	Y	N	0.4	Y	N	Y	N	Y	0.6	Y	Y	N	Y	Y	0.8	Y	Y	Y	Y	Y	1
13	Y	Y	Y	Y	N	0.8	Y	Y	Y	Y	Y	1	Y	Y	N	N	Y	0.6	N	Y	Y	N	Y	0.6
14	Y	Y	N	Y	N	0.6	N	Y	Y	Y	Y	0.8	Y	Y	Y	Y	N	0.8	N	Y	Y	Y	N	0.8
15	Y	Y	N	Y	Y	0.8	Y	Y	Y	N	Y	0.8	Y	Y	Y	Y	N	0.8	N	Y	Y	N	Y	0.6
16	N	Y	N	N	N	0.2	Y	N	Y	Y	Y	0.8	Y	N	Y	Y	Y	0.8	Y	Y	N	Y	Y	0.8
17	N	N	Y	N	N	0.2	N	Y	Y	Y	N	0.6	Y	Y	N	Y	Y	0.8	Y	Y	Y	N	Y	0.8
18	Y	N	Y	N	Y	0.6	N	Y	Y	Y	Y	0.8	Y	N	Y	Y	Y	0.8	Y	Y	Y	Y	Y	1
19	N	N	N	Y	Y	0.4	Y	Y	Y	Y	N	0.8	Y	N	Y	N	Y	0.6	Y	Y	Y	Y	Y	1
20	N	N	Y	N	Y	0.4	Y	Y	Y	N	Y	0.8	Y	Y	Y	Y	Y	1	Y	Y	Y	N	Y	0.8

Suppl. Table S3, (A): Avoidance index to 0.5M glycerol drop test in worms of four studied groups (Control: untreated animals, VitC: worms grown at the presence of 1mM vitamin C, PQ: worms grown at the presence of 0.1mM PQ, VitC/PQ: worms grown at the simultaneous presence of 1 mM vitamin C and 0.1mM PQ), for Day 1 worms (L4 animals are considered as Day 0 worms). 20 worms assayed, 5 trials/worm. Y: positive response (avoidance), N: negative response (no avoidance).

SUPPL. TABLE 3, Glycerol Avoidance Index, (B) Day4

	Day4																					
Worm	Control					A.I.	PQ					A.I.	VitC					A.I.	VitCPQ					A.I.
1	Y	Y	N	N	Y	0.6	Y	Y	Y	Y	N	0.8	N	Y	Y	N	Y	0.6	Y	N	N	Y	Y	0.6
2	Y	N	Y	Y	N	0.6	Y	N	Y	Y	Y	0.8	C	N	N	Y	Y	0.6	N	Y	Y	Y	N	0.6
3	N	Y	N	N	Y	0.4	N	Y	Y	Y	Y	0.8	N	Y	Y	Y	N	0.6	N	N	N	Y	Y	0.4
4	N	Y	Y	Y	Y	0.8	Y	Y	N	N	Y	0.6	N	Y	N	N	Y	0.4	C	Y	N	Y	N	0.6
5	N	N	Y	Y	N	0.4	Y	Y	Y	Y	N	0.8	Y	N	Y	Y	N	0.6	Y	N	Y	Y	N	0.6
6	Y	Y	Y	N	Y	0.8	Y	N	N	Y	Y	0.6	N	N	N	N	Y	0.2	Y	Y	N	N	Y	0.6
7	N	N	Y	Y	Y	0.6	Y	Y	Y	N	Y	0.8	N	Y	Y	N	Y	0.6	N	N	Y	Y	N	0.4
8	Y	Y	N	Y	Y	0.8	Y	N		Y	Y	0.75	Y	N	N	Y	N	0.4	N	Y	Y	Y	N	0.6
9	N	N	Y			0.33	Y	Y	N	N	Y	0.6	N	Y	N	N	Y	0.4	Y	N	N	Y	Y	0.6
10	Y	Y	N			0.66	Y	Y	Y	Y	N	0.8	N	Y	N	Y	Y	0.6	N	N	Y	Y	N	0.4
11	N	Y				0.5	N	Y	Y	Y	Y	0.8	N	Y	Y	N	Y	0.6	Y	N	Y	N	Y	0.6
12	Y	Y	N	Y	N	0.6	Y	N	Y	Y	Y	0.8	N	Y	Y	Y	N	0.6	Y	N	Y	N	N	0.4
13	N	N	Y	Y	Y	0.6	N	Y	Y	Y	Y	0.8	Y	N	Y	Y	N	0.6	Y	Y	Y	N	N	0.6
14	Y	Y	N	Y	Y	0.8	Y	Y	N	N	Y	0.6	Y	N	N	Y	N	0.4	Y	N	Y	Y	N	0.6
15	N	Y	Y	Y	N	0.6	Y	Y	Y	N	Y	0.8	N	Y	Y	N	Y	0.6	N	N	N	Y	Y	0.4
16	Y	Y	N	N	N	0.4	Y	N	N	Y	Y	0.6	N	N	Y	N	N	0.2	Y	Y	N	N	Y	0.6
17	N	N	Y	Y	N	0.4	Y	Y	Y	Y	N	0.8	N	Y	Y	N	Y	0.6	Y	N	N	Y	Y	0.6
18	Y	Y	Y	N	Y	0.8	Y	Y	Y		N	0.75	Y	N	N	Y	N	0.4	Y	N	Y	Y	N	0.6
19	Y	N	Y	N	N	0.4						0.6						0.6	Y	N	N	Y	N	0.4
20	N	Y	N	Y	Y	0.6	Y	Y	Y	Y	N	0.8	Y	Y	N	N	N	0.4	Y	N	N	Y	Y	0.6

Suppl. Table S3, (B): Avoidance index to 0.5M glycerol drop test in worms of four studied groups (Control: untreated animals, VitC: worms grown at the presence of 1mM vitamin C, PQ: worms grown in the presence of 0.1 mM PQ, VitC/PQ: worms grown in the simultaneous presence of 1 mM vitamin C and 0.1mM PQ), for Day 4 worms (L4 animals are considered as Day 0 worms). 20 worms assayed, 5trials/worm. Y: positive response (avoidance), N: negative response (no avoidance).

SUPPL. TABLE 3, Glycerol Avoidance Index, (C) Day5

	**Day5**																							
Worm	Control					A.I.	PQ					A.I.	VitC					A.I.	VitCPQ					A.I.
1	N	Y	N	Y	Y	0.6	Y	Y	N	Y	Y	0.8	Y	N	Y	Y	Y	0.8	Y	N	Y	N	Y	0.6
2	N	Y	Y	N	N	0.4	Y	N	Y	N	N	0.4	N	Y	N	N	Y	0.4	N	Y	N	N	N	0.2
3	Y	N	N	Y	N	0.4	N	Y	N	Y	Y	0.6	Y	Y	Y	Y	N	0.8	N	Y	N	Y	Y	0.6
4	N	Y	Y	N	Y	0.6	Y	Y	Y	Y	N	0.8	Y	N	N	N	Y	0.4	Y	N	Y	Y	Y	0.8
5	N	N	Y	N	N	0.2	N	N	Y	Y	Y	0.6	N	Y	Y	Y	N	0.6	N	Y	N	N	N	0.2
6	Y	Y	N	Y	Y	0.8	N	Y	N	Y	N	0.4	Y	N	N	Y	Y	0.6	N	N	Y	N	Y	0.4
7	N	N	Y	N	N	0.2	Y	N	Y	Y	Y	0.8	N	Y	Y	N	Y	0.6	Y	N	Y	Y	Y	0.8
8	Y	N	Y	Y	Y	0.8	N	Y	N	N	N	0.2	Y	Y	Y	Y	N	0.8	N	Y	Y	N	N	0.4
9	N	Y	N	Y	Y	0.6	Y	N	Y	Y	Y	0.8	N	N	Y	N	Y	0.4	N	Y	N	Y	Y	0.6
10	N	Y	Y	N	N	0.4	N	Y	Y	N	Y	0.8	Y	N	N	Y	Y	0.6	N	Y	Y	N	N	0.4
11	Y	N	N	Y	N	0.4	Y	N	Y	Y	Y	0.8	N	Y	Y	Y	N	0.6	Y	Y	Y	Y	N	0.8
12	Y	Y	Y	N	N	0.6	N	Y	Y	N	N	0.4	N	Y	Y	N	N	0.4	N	Y	Y	Y	N	0.6
13	N	Y	N	Y	Y	0.6	N	Y	Y	Y	N	0.6	Y	N	Y	Y	Y	0.8	Y	N	N	N	Y	0.4
14	N	Y	Y	N	N	0.4	Y	Y	Y	Y	N	0.8	N	N	N	Y	N	0.4	N	Y	N	Y	Y	0.6
15	Y	N	N	Y	N	0.4	N	Y	N	Y	Y	0.6	Y	Y	Y	Y	N	0.8	Y	N	N	N	N	0.2
16	Y	N	Y	N	Y	0.6	N	Y	Y	N	N	0.4	N	Y	Y	N	N	0.4	Y	Y	Y	N	N	0.6
17	N	N	N	N	Y	0.2	Y	N	Y	Y	Y	0.8	N	Y	N	Y	Y	0.6	N	Y	N	Y	Y	0.6
18	N	Y	Y	Y	Y	0.8	N	Y	N	N	N	0.2	N	Y	Y	Y	N	0.6	Y	N	Y	N	N	0.4
19	N	N	Y	N	N	0.2	Y	Y	Y	N	Y	0.8	N	Y	N	Y	Y	0.6	Y	Y	Y	Y	N	0.8
20	Y	Y	N	Y	Y	0.8	Y	N	Y	Y	Y	0.8	Y	Y	Y	Y	N	0.8	N	Y	N	N	N	0.2

Suppl. Table S3, (C): Avoidance index to 0.5M glycerol drop test in worms of four studied groups (Control: untreated animals, VitC: worms grown at the presence of 1mM vitamin C, PQ: worms grown in the presence of 0.1mM paraquat, VitC/PQ: worms grown in the simultaneous presence of both 1mM vitamin C and 0.1mM paraquat), for Day 5 worms (L4 animals are considered as Day 0 worms). 20 worms assayed, 5trials/worm. Y: positive response (avoidance), N: negative response (no avoidance).

SUPPL. TABLE 4, Locomotion Parameters Data, (A) Velocity

VELOCITY (um/sec)											
**Day1**				**Day4**				**Day5**			
Control	VitC	PQ	VitCPQ	Control	VitC	PQ	VitCPQ	Control	VitC	PQ	VitCPQ
71.1	100.76	45.96	65	78.78	96.98	130.1	27.7	71.5	37.88	26.16	52.64
83	13.06	66	81.54	84.5	62.4	119.36	58.56	68.18	101.78	39.64	46.14
81.76	78.76	89.42	73.82	39.2	57.52	83.2	52.94	43.68	47.5	53.06	56.62
57.78	99.3	80.74	36.74	74.86	63.26	68.52	75.72	87.16	56	57.74	55.6
71.82	8.36	57.68	84.88	61.84	92.36	51.32	27.08	101.18	71.82	36.12	58.02
75.24	102.62	98.5	140.74	54.94	57.26	69.6	11.54	85.28	86.24	36.76	45.14
70.68	50.88	81.92	66.84	80.3	90.7	97.78	73.44	77.88	87.3	39.78	49.8
70.4	80.96	88.26	79.12	91.82	60.38	86.4	96.38	137.18	69.98	50.66	40.28
91.8	48.5	71.28	65.9	73.34	84.84	53.94	103.88	105.48	65.41	68.42	53.18
74.58	15.67	80.28	80.54	127.14	93.86	87.24	81.44	56.06	70.82	53.18	56.03
48.44	10.36	100.4	85.8	73.7	94.45	71.14	93.66	111.78	61.89	31.68	58.21
70.26	100.4	75.2	28.23	52.04	60.54	63.18	64.9	92.3	67.89	46.36	44.98
59.96	98.9	49.5	66.1	73.32	100.9	91.8	98.42	123.02	71.59	37.94	54.6
67.96	83.1	32.76	100.3	37.76	59.26	72.28	58.9	58.76	77.6	51.4	53.78
66.62	100.45	72.97	81.3	71.46	58.36	65.06	73.98	63.3	25.1	45.26	47.16
86.56	77.8	80.92	80.82	56.78	60.01	86.4	30.25	82.16	95.9	45.92	40.35
71.5	68.1	64.21	36.2	98.8	94.7	74.7	62.7	61	89	46.64	50.9
74.3	65.9	60.11	150.1	49.86	56.4	79.3	46.2	59.64	70.3	49.16	51.9
		74.23		39.2	84.6			68.3			
								68.18			

Suppl. Table S4, (A) Velocity (um/sec) of freely moving worms of three different ages (Control: untreated animals, VitC: worms grown at the presence of 1mM vitamin C, PQ: worms grown in the presence of 0.1mM PQ, VitC/PQ: worms grown in the simultaneous presence of both 1mM vitamin C and 0.1mM PQ, L4 animals are considered as Day 0 worms). 18-22 worms were tracked from each studied group.

SUPPL. TABLE 4, Locomotion Parameters Data, (B) Bending Amplitude

**BENDING AMPLITUDE (mm)**											
**Day1**				**Day4**				**Day5**			
Control	VitC	PQ	VitCPQ	Control	VitC	PQ	VitCPQ	Control	VitC	PQ	VitCPQ
0.07	0.12	0.08	0.08	0.13	0.09	0.09	0.09	0.11	0.09	0.11	0.09
0.09	0.14	0.08	0.1	0.11	0.11	0.08	0.21	0.12	0.07	0.11	0.12
0.08	0.11	0.09	0.12	0.15	0.11	0.1	0.08	0.13	0.08	0.07	0.07
0.08	0.11	0.08	0.12	0.11	0.1	0.11	0.11	0.19	0.09	0.1	0.09
0.08	0.52	0.08	0.08	0.11	0.11	0.08	0.12	0.07	0.09	0.08	0.08
0.08	0.09	0.08	0.07	0.13	0.12	0.12	0.1	0.09	0.07	0.11	0.09
0.08	0.12	0.14	0.11	0.12	0.09	0.1	0.1	0.08	0.1	0.11	0.09
0.08	0.13	0.08	0.09	0.11	0.08	0.14	0.11	0.08	0.09	0.11	0.07
0.1	0.05	0.13	0.08	0.13	0.07	0.1	0.08	0.09	0.08	0.14	0.11
0.11	0.15	0.1	0.07	0.12	0.08	0.15	0.09	0.11	0.09	0.09	0.13
0.14	0.67	0.11	0.06	0.09	0.08	0.14	0.09	0.24	0.09	0.11	0.09
0.14	0.11	0.09	0.08	0.1	0.09	0.09	0.01	0.19	0.07	0.09	0.12
0.14	0.12	0.17	0.1	0.08	0.07	0.08	0.11	0.08	0.08	0.12	0.07
0.12	0.11	0.19	0.09	0.07	0.09	0.11	0.11	0.1	0.09	0.1	0.09
0.11	0.15	0.1	0.1	0.09	0.08	0.09	0.09	0.1	0.09	0.11	0.09
0.13	0.14	0.1	0.09	0.13	0.09	0.11	0.1	0.13	0.07	0.07	0.09
0.09	0.1	0.11	0.1	0.17	0.1	0.09	0.1	0.13	0.1	0.11	0.12
0.1	0.08	0.09	0.1	0.11	0.08	0.07	0.11	0.14	0.09	0.12	0.09
				0.15				0.11		0.1	
				0.01				0.11			
				0.12				0.09			

Suppl. Table S4, (B): Bending amplitude (mm) of freely moving worms of three different ages (Control: untreated animals, VitC: worms grown at the presence of 1mM vitamin C, PQ: worms grown at the presence of 0.1 mM PQ, VitC/PQ: worms grown in the simultaneous presence of 1 mM vitamin C and 0.1 mM PQ, L4 animals are considered as Day 0 worms). 18-22 worms were tracked from each studied group.
